# Regulatory compliance among over-the-counter medicine sellers facilities within the Upper East Region of Ghana

**DOI:** 10.1186/s40545-021-00363-2

**Published:** 2021-08-24

**Authors:** Benjamin Kwarteng Frempong, Anthony Amalba, Nina Donkor, Kwadwo Owusu Akuffo

**Affiliations:** 1 Pharmacy Council, Upper East Region, Bolgatanga, Ghana; 2 Department of Health Professions Education and Innovative Learning, School of Medicine, University for Development Studies, Tamale, Ghana; 3 Department of Pharmacy Practice, College of Health Sciences, Kwame Nkrumah University of Science and Technology, Kumasi, Ghana; 4 Department of Optometry and Visual Science, College of Science, Kwame Nkrumah University of Science and Technology, Kumasi, Ghana

**Keywords:** Over-the-counter medicine sellers, Regulatory compliance, Upper East Region, Ghana

## Abstract

**Background:**

Easy access to medicines provided by private medicine retailing facilities including that of over-the-counter medicine retailers, have gained prominence in sub-Saharan Africa. Although over-the-counter medicine-sellers (OTCMS) facilities play an indispensable role in healthcare delivery, there is inadequate information about their regulatory environment and whether their operations conform to regulatory provisions. Hence, this study sought to investigate the characteristics and predictors of regulatory practices among over-the-counter medicine sellers in Ghana.

**Methods:**

This was a cross-sectional study involving participants from 208 OTCMS facilities in eight (8) municipalities and districts (MDA’s) of the Upper East Region of Ghana. An initial census of facilities in the region was conducted between May and August 2016 and a follow-up conducted between December 2016 and March 2017. This ensured the identification and location of all OTCMS facilities within the selected MDA’s for study planning and data collection. The main outcome variable was regulatory compliance which is a composite of three indicators for regulatory practices (retention of medicine supplier’s invoices and receipts on-premises), licensing and registration requirements (appropriate signage), and equipment and material requirements (availability of reference material). Regulatory compliance was assessed using bivariate and multivariate logistic regression analyses.

**Results:**

In this survey, 21.5%, 38.2%, and 23.1% of the facilities surveyed had a good state of repair, had the owner of the facility available on the premises, and had received regulatory visit(s) in less than 12 months, respectively. Only 29.2% of facilities were regulatory compliant. After statistical adjustment, OTCMS facility location (compared with Rural: Urban, AOR = 4.2, 95% CI 1.74–10.17, *p* = 0.001) and staff trained in less than 1 year (AOR = 2.78, 95% CI 1.02–7.62, *p* = 0.046) were significantly associated with regulatory compliance.

**Conclusions:**

Regulatory compliance was low in the Upper East Region of Ghana, particularly across rural locations, where most of the facilities failed to meet the laid down provisions of the Pharmacy Council regarding practice, staff and premises requirements. This could be attributed to the fact that these areas are poorly resourced. Policymakers are been called on to put in place pragmatic measures in relation to OTCMS facility’s location and regulatory requirements to address the inequities in compliance.

## Background

Private medicine retail outlets, including over-the-counter medicine seller facilities, are recognized as major stakeholders in the healthcare sector globally, especially in developing and under-developed countries [1–3]. Currently, they are acknowledged as treatment centres for the management of diseases of common occurrence including, but not limited to malaria [3–6], diarrhoea [7] and respiratory infections [8–10]. They are the first point of call for the treatment of diseases in most low- and middle-income countries [11, 12]. Consequently, placing a growing demand on policymakers to incorporate their vital role in the development of health policies are aimed at enhancing access to services and improving public health [13, 14]. The increasing demand for the services of these private healthcare providers could be attributed to a host of factors such as accessibility, suitability, prompt service delivery, flexibility in payment terms, as well as flexible purchasing options and operational hours [11, 15–18]. The operations of private medicine retailers have been further heightened by the relatively short waiting hours and affordable services as compared to receiving treatment in hospitals and clinics [19, 20].

The scope of private medicine retailers has been shown to differ from one country to another [21] though they trade mostly in drugs, drug-related commodities, and non-drug items. In Ghana, private medicine retailers include community pharmacies which may be pharmacist, non-pharmacist or partly-pharmacist owned and over-the-counter medicine seller (OTCMS) facilities.

The main law that regulates Pharmacy practice in Ghana is the Health Professions Regulatory Bodies Act, Act 857, 2013 though some other regulations are also enforceable within the pharmaceutical sector (Table 1). Inspecting officers from these regulatory bodies carry out regulation of the activities of pharmacies and over-the-counter medicine seller facilities and on such visits, they conduct routine checks on the premises and offer technical and supportive advice to practitioners or available staff (Table 2). OTCMS facilities are authorized to retail drugs other than prescription-only medicines or pharmacy-only medicines, in rural and peri-urban settings where pharmacists are not readily available to render pharmaceutical service [22–24]. They are restricted to the sale of over-the-counter medicines: drugs that are generally regarded as safe for the consumer for use by following the required label directions and warnings. They may be purchased without a prescription. They are expected to meet the minimum regulatory requirements set for private healthcare providers [24, 25] as well as standards prescribed for medicine retail outlets [26, 27].

**Table 1:** Laws governing pharmaceutical retail sector in Ghana

Legislation	Main purpose	Frontline officers
Part IV of the Health Professions Regulatory Bodies Act, Act 857, 2013	Establishes Pharmacy Council of Ghana (PC) as a statutory regulatory body to secure in the public interest the highest standards in the practice of pharmacy in Ghana	Inspecting Pharmacists of the Pharmacy Council of Ghana
Public Health Act, 2012, Act 851	Establishes the Food and Drugs Authority to provide standards for the sale of food and drugs and for related matters. Part 7 & 8 of Act 851 regulates medicines to ensure that they are safe, efficacious and of right quality	Inspecting Officers of the Ghana Food and Drugs Authority (FDA)
Health Institutions and Facilities Act, 2011, Act 829	Establishes the Health Facilities Regulatory Agency (HEFRA) to license facilities for the provision of public and private health care services (premises license)	Officers from HEFRA and the Ghana Health Service (GHS)

**Table 2:** Summary of regulatory requirements for a medicine retail outlet in Ghana

Standards	Some specific requirements
Premises/structure	Must be geographically and structurally permanent Premises must be fit for purpose intended/well designed (ceiling intact, shelves, floor, counter, walls & painting, hygiene, layout etc.); adequately illuminated and ventilated, storage facilities provided, appropriate customer and dispensing areas etc Premises must meet minimum requirements for floor space and ceiling height
Equipment and materials	Availability of a reference material Availability of ancillary devices (weighing scales, thermometers etc.) where applicable
Personnel	Must not be a minor (must be ≥ 18 years of age) Must be educated (at least SHS level for OTCMS, dispensing/counter assistants) Must undergo periodic training and re-training
Practice-related	Annual renewal of premises and operating licenses Ensure good records keeping Premises and business operating license must be displayed Ensure good dispensing and storage practices are adhered to Must have a well-written signboard Premises shall only be employed for the purpose intended or for which operating license was issued (range of services, range of products/commodities in stock, class of drug products in stock) Only drugs of the approved class shall be stocked and sold where applicable

It is, however, perennial to see such prescribed standards being flouted. Documented regulatory offences include the sale of substandard or fake drugs, non-compliance to set standards for personnel/premises, operating without due authorization or valid license as well as the sale of prescription-only drugs [24–27]. These regulatory offences have the potential to undermine public confidence in these facilities or healthcare providers as they compromise the quality of care and service delivery.

Given the scarcity of health professionals including pharmacists in the hinterlands and the consequent deprivation of under-developed regions of the services of a pharmacist [12], over-the-counter medicine seller facilities have been licensed in order to reduce the discrepancies in access to health care delivery in such areas [24]. OTCMS facilities are thus predominant in the Upper East Region and remain the first port of call for most of its inhabitants. Considering the increasing patronage of the services of over-the-counter medicine sellers in the region, investigating whether these service providers practice within agreed standards will provide important data on regulatory compliance with potential implications for public health interventions. This study sought to assess the compliance of OTCMS facilities within selected municipalities and districts (MDA’s) of the Upper East Region of Ghana to selected prescribed regulations and to identify the possible factors associated with non-compliance.

## Methods

### Study design

An initial census of facilities in the Upper East Region of Ghana was conducted between May and August 2016 and a follow-up was conducted between December 2016 and March 2017 to ensure that all the OTCMS facilities within the selected MDA’s were located for study planning and data collection.

### Study population

**Fig. 1:**
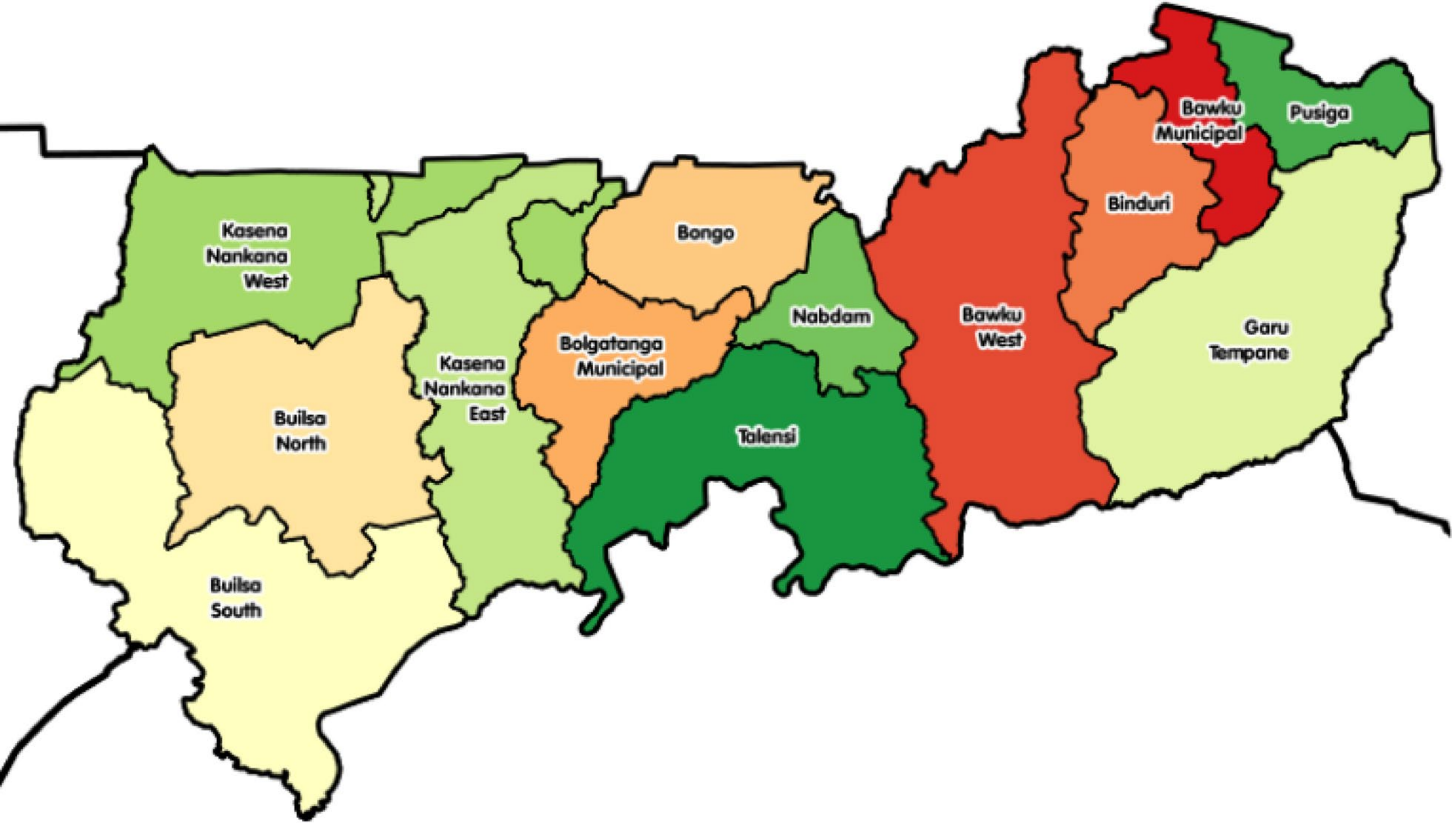
Map of the Upper East region of Ghana showing the study Municipalities and Districts

In this cross-sectional study, eight (8) out of 13 MDA’s of the Upper East Region of Ghana (Fig. 1) were selected; namely: Bolga Municipal, Bongo district, Kassena Nankana Municipal, Kassena Nankana West district, Bawku Municipal, Garu-Tempane district, Bawku West district and Pusiga district. According to the 2010 housing and population census in Ghana, these 8 districts/municipalities were among the most densely populated areas within the Upper East Region [28]. Furthermore, these districts were selected because data from the Pharmacy Council of Ghana (the main regulatory body of the pharmacy profession in Ghana) showed that majority of OTCMS facilities were found in these areas. In addition, the selected areas had distinct urban and rural locations, which allowed outcomes to be compared across rural/urban settings.

The Upper East Region has a total land surface area of 8842 km^2^ and covers about 2.7% of the total land surface area of Ghana. The population density of the region as of the year 2010 was 118.4/km^2^ [28]. The predominant occupation in the region is agriculture with 65.9% of the populace of the region engaged in agriculture and its related work [29].

In terms of health, the region is divided into 13 administrative districts and 86 health sub-districts. The region boasts of 297 health facilities (excluding private medicine retail outlets) and 89% of these facilities are under the administration of the Upper East Regional Health Directorate. Of the 297 health facilities, the government/state owns 266, 18 are privately owned, 12 are mission-owned and 1 is a quasi-government facility. Interestingly, only 60% of the population is within an 8 km radius of these health facilities. These facilities, however, face serious staff challenges and the doctor-to-patient ratio in these facilities woefully falls below the World Health Organization (WHO) recommended ratio of 1:1000 [30]*.*

### Sampling and sample size

A universal sampling technique was employed, and thus, all facilities identified in the selected districts were included in the study. A total number of 208 OTCMS facilities were identified across the 8 selected MDA. Out of this number, 22 of the facilities refused to participate, thus response was obtained for 186 participants.

### Data collection

#### Selection of OTCMS facilities and participants

During the census survey, OTCMS facilities were identified by three complementary approaches. The first strategy was to employ demographic and public health staff resident in the selected areas through the various district and municipal health directorates. The second strategy was to use facility owners or staff in identified shops to identify other retail outlets within the area. The last approach was the recruitment of a field team in the various districts that facilitated facility identification. Over-the-counter medicine seller outlet was defined as facilities not regarded as a community pharmacy, operated outside a hospital, clinic, health centre or Community-based Health Planning and Services (CHPS) zone and satisfied the premises/structure requirement of being geographically and structurally permanent and being fit for the intended purpose. The census survey showed that drugs were not only available for sale in OTCMS facilities, but also, on tables and trucks in market and lorry stations and kiosks. OTCMS (like community pharmacies and drug outlets) that operated within hospitals, clinics, health centres and CHPS zones and therefore were excluded from the study.

### Data collection tool and procedures

Data were collected from April to August 2017 with the aid of a carefully designed survey tool. The survey tool covered areas such as location/setting of the facility (rural versus urban), frequency of inspection visits, interactions/associations with regulatory officers, personnel/staff characteristics, premises-related features, equipment and materials, and practice-related characteristics. The facilities identified through the census of facilities served as a sampling frame for the study. The survey tool was first piloted outside the selected districts for consistency and re-shaping. Following verbal consent, the questionnaires were administered to front-line staff (owner or staff) involved in the daily management of the facilities. Questionnaire administration was carried out by 9 field-trained staff and the medium of communication was mainly English language since the staff of OTCMS facilities were often considered to have some level of formal education. The survey tool (which takes 20–30 min to administer) had two sections: the first section involved a structured questionnaire which contained a series of questions read to front-line staff for responses and the second section deployed the use of an observation checklist to assess compliance of the facility to some selected regulatory practices. The trained field staff only used the checklist to assess whether facilities included in the study complied with selected premises and practice regulations or not.

To provide a vivid understanding of the regulatory environment, regulatory interactions were also assessed. The Demographic and Public Health Staff recruited played a vital role during data collection by constantly assuring practitioners or staff that data collectors were not associated with any of the regulatory agencies. A strategy employed to maintain a congenial atmosphere was to ask sensitive regulatory questions towards the end of the interview after good rapport had been established with the service providers and they were much more settled. This allowed the free flow of information needed for the study. Of note, the sole aim of this study was to assess regulatory compliance and not the relative significance of one regulation over another.

### Data management and analysis

Data were double entered in Microsoft Access 2013 and analysed using Statistical Package for Service Solution (SPSS, IBM Software version 16.0, Chicago, USA). The unit of analysis is over-the-counter medicine sellers (individual participants). Descriptive statistics were used to determine the frequencies and percentages of demographic characteristics.

For the study and analysis, facilities operating within the town and less than 3 km from town were classified as being sited in urban locations. Those sited 3 km or more away from town were regarded as rural facilities.

The main outcome variable is Regulatory Compliance which is a composite of three indicators for regulatory practices (retention of medicine supplier’s invoices and receipts on-premises), licensing and registration requirements (appropriate signage), and equipment and material requirements (availability of reference material).

To investigate associations with regulatory compliance, bivariate logistic regression was first conducted. This was then followed by multivariate logistic regression with only variables with a univariate Wald test *p*-value of < 0.2 considered for inclusion in the multivariate model. For all analysis, *P*-value ≤ 0.05 is considered statistically significant. The study aimed to compare regulatory compliance across the MDA’s as well as to determine the predictors of regulatory compliance and not to assess the influence of individual MDA’s on regulatory compliance.

## Results

### Characteristics and regulatory practices of over-the-counter medicine seller facilities

A total number of 208 OTCMS facilities were identified across the 8-selected MDA (Bawku Municipal (*n* = 30), Garu-Tempane district (*n* = 29), Kassena Nankana West district (*n* = 24), Kassena Nankana Municipal (*n* = 23), Bongo district (*n* = 21), Pusiga district (*n* = 13), Bawku West district (*n* = 13) and Bolga Municipal (*n* = 55). The number of OTCMs facilities identified exceeded the estimated figure of 176 OTCMS facilities provided by the Pharmacy Council (the main Regulator) across the selected districts. Twenty-two (10.58%) of staff out of the total number of 208 identified OTCMS facilities refused to consent to the institutional review board (IRB) approved protocol for the study, and hence, were excluded. Overall, the study response rate was 89.42% (186). The major factor accounting for the refusals was the absence of main staff during the period of the survey. Of the 186 respondents, 115 (61.8%) of the respondents were shop assistants and 71 (38.2%) were shop owners. Over 50% of the respondents had an educational background above junior high school with only 0.5% having no formal educational background.

The majority, 101(54.3%) of the OTCMS facilities involved in the survey, were located in the urban areas; whereas, 85 OTCMS (45.7%) were situated in the rural areas.

Table 3 shows selected regulatory characteristics of OTCMS and regulatory interactions across the selected MDA’s and rural–urban locations. Lowest compliance was found on the knowledge of staff of the name of the law that regulates pharmacy practice, state of repair and the durability of floor and ease of cleaning. With regard to premises related characteristics, the size of the dispensing area and design of the dispensing area were found to comply with regulations. Compliance to this preamble across the eight selected MDA’s was similar and high.

**Table 3:** Regulatory environment interactions and regulatory characteristics of OTCMS facilities across selected MDAs and rural–urban locations (*n* = 186)

Variables	Municipalities and districts (MDA’S) Facilities^f^									Location		
	BM (*N* = 25)	GT (*N* = 25)	KNW (*N* = 23)	KNM (*N* = 19)	Bo (*N* = 20)	P (*N* = 11)	BW (*N* = 11)	B (*N* = 52)	*p*-value	Urban (*N* = 101)	Rural (*N* = 85)	*p*-value
	*n* (%)	*n* (%)	*n* (%)	*n* (%)	*n* (%)	*n* (%)	*n* (%)	*n* (%)		*n* (%)	*n* (%)	
Premises characteristics												
State of repair	7 (28.0)	1 (4.0)	2 (8.7)	7 (36.8)	4 (20.0)	0 (0.0)	3 (27.3)	16 (30.8)	0.0026	31( 30.7)	9 (10.6)	0.001
Durability of floor and ease of cleaning	14 (56.0)	6 (24.0)	10 (43.5)	13 (68.4)	3 (15.0)	2 (18.2)	3 (27.3)	33 (63.5)	< 0.001	61 (60.4)	25 (27.1)	0.001
Size of dispensing area appropriate	19 (76.0)	23 (92.0)	20 (87.0)	17 (89.5)	15 (75.0)	9 (81.9)	10 (90.9)	43 (82.7)	0.706	84 (83.2)	72 (84.7)	0.776
Design of dispensing area appropriate	23 (92.0)	25 (100.0)	19 (82.6)	18 (94.7)	19 (95.0)	8 (72.7)	11 (100.0)	44 (84.6)	0.114	90 (89.1)	77 (90.6)	0.74
Staff characteristics												
Owner of facility available on premises	9 (36.0)	13 (52.0)	10 (43.5)	6 (31.6)	9 (45.0)	4 (36.4)	2 (18.2)	18 (36.4)	0.629	38 (37.6)	33 (38.8)	0.867
Staff trained in less than a year	21 (84.0)	13 (52.0)	13 (56.5)	10 (52.6)	13 (65.0)	6 (54.6)	7 (63.6)	34 (65.4)	0.35	71 (70.3)	46 (54.1)	0.023
Staff knowledge on regulatory law^a^	0 (0.0)	1 (4.0)	0 (0.0)	0 (0.0)	0 (0.0)	0 (0.0)	0 (0.0)	2 (3.8)	0.34	2 (2.0)	1 (1.2)	0.586
Material and equipment characteristics												
Reference books available on premises	13 (52.0)	13 (52.0)	11 (47.8)	8 (42.1)	8 (40.0)	4 (36.4)	4 (36.4)	28 (53.8)	0.885	52 (51.5)	37 (43.5)	0.279
Practice-related characteristics												
Retention of supplier's invoices and receipts	17 (68.0)	10 (40.0)	18 (78.3)	9 (47.1)	11 (27.3)	3 (27.3)	5 (45.5)	39 (75.0)	0.005	69 (68.3)	37 (43.5)	0.002
Appropriate signboard mounted	22 (88.0)	10 (40.0)	8 (34.8)	6 (31.6)	10 (50.0)	6 (54.5)	4 (36.4)	39 (75.0)	< 0.001	68 (67.3)	37 (43.5)	0.002
Stocking POM (Class A)	21 (84.0)	23 (92.0)	22 (95.7)	18 (94.7)	20 (100.0)	11 (100.0)	11 (100.0)	49 (94.2)	0.376	95 (94.1)	80 (94.1)	0.987
Stocking Pharmacy medicines (Class B)	25 (100.0)	25 (100.0)	23 (100.0)	19 (100.0)	20 (100.0)	11 (100.0)	11 (100.0)	52 (100.0)		101 (100.0)	85 (100.0)	
Stocking of non-drug items (e.g., Cotton)	22 (88.0)	19 (76.0)	18 (78.3)	16 (84.2)	19 (95.0)	7 (63.6)	10 (90.9)	51 (98.1)	0.019	92 (91.1)	70 (82.4)	0.077
Stocking of household items	0 (0.0)	0 (0.0)	0 (0.0)	0 (0.0)	0 (0.0)	0 (0.0)	0 (0.0)	0 (0.0)		0 (0.0)	0 (0.0)	
Stocking of herbal remedies	18 (72.0)	16 (64.0)	17 (73.9)	14 (73.7)	19 (95.0)	10 (90.9)	8 (72.7)	36 (69.2)	0.313	80 (79.2)	58 (68.2)	0.088
Frequency of regulatory visits												
Regulatory visit in less than a year	2 (8.0)	2 (8.0)	0 (0.0)	0 (0.0)	0 (0.0)	0 (0.0)	2 (18.2)	37 (71.2)	< 0.001	36 (35.6)	7 (8.2)	0.001
No regulatory visits	1 (4.0)	5 (20.0)	4 (17.4)	0 (0.0)	7 (35.0)	2 (18.2)	0 (0.0)	5 (9.6)	< 0.001	6 (5.9)	18 (21.2)	0.001
Regulatory interactions												
Ever received prior notification^b^	22 (88.0)	8 (32.0)	13 (56.5)	12 (63.2)	10 (50.0)	5 (45.5)	1 (9.1)	27 (51.9)	< 0.001	62 (61.4)	36 (42.4)	0.01
Prior knowledge of identity^c^	10 (40.0)	3 (12.0)	12 (52.2)	7 (36.8)	8 (40.0)	1 (9.1)	13 (25.0)	58 (31.2)	0.043	36 (35.6)	22 (25.9)	0.152
Establishing close relationships important^d^	19 (76.0)	21 (84.0)	21 (91.3)	19 (100.0)	19 (95.0)	8 (72.7)	8 (72.7)	41 (78.8)	0.159	84 (83.2)	72 (84.7)	0.776
Ever contributed in cash or kind^e^	16 (64.0)	8 (32.0)	9 (39.1)	7 (36.8)	10 (50.0)	5 (45.5)	0 (0.0)	9 (17.3)	0.001	31 (30.7)	33 (38.8)	0.245

^a^Includes staff who could correctly name the Health Professions Regulatory Bodies Act, Act 857, 2013 as the main legislation that governs pharmacy practice

^b^Includes staff who had ever received prior notification before regulatory visits by Inspecting Pharmacists either through Pharmacy Council staff, the staff of other OTCMS facilities or any other means

^c^Includes staff who had ever known the identity/name of the Inspection Officer assigned for routine checks before the arrival of that particular officer

^d^Includes staff who thought it was important to establish a close relationship/rapport with Inspecting Officers/other Pharmacy Council staff/Pharmacy Council Office for various reasons

^e^Includes staff who had ever made any contribution or donation in cash/kind either directly or through other(s) to Inspecting Officers/Pharmacy Council staff/Pharmacy Council Office to establish relationships or for any other reason

^f^Includes all selected MDA’S: BM, Bawku Municipal; GT, Garu-Tempane; KNW, Kassena Nankana West; KNM, Kassena Nankana Municipal; Bo, Bongo; P, Pusiga; BW, Bawku west district

Compliance with selected staff characteristics was relatively low across the eight-selected MDA’s. The regulation “staff knowledge of the name of the law that regulates pharmacy practice” was similar across the MDA’s. Relatively few OTCMS facilities (38.2%) had the facility owners available on the premises during the survey. Compliance with this staff characteristic was relatively lower in the municipalities than in the districts. Less than 53% of the facilities in the rural areas had the owners present. Also, over 45% of main staff in OTCMS facilities across all the selected MDA’s and rural–urban locations had undergone re-training in the past 1-year.

For material characteristics, the availability of reference material was relatively low and variable across the 8 selected MDA’s.

The practice-related characteristic that recorded the highest non-compliance across the selected MDA’s was stocking of prescription-only medicines and it was very high and comparable across the 8 MDA’s. The availability of medicine supplier’s invoices and receipts was variable, and it was the practice-related characteristic that recorded the highest compliance. The availability of an appropriately mounted signboard varied across the 8 MDA’s from the lowest percentage of 31.6% (Kassena Nankana Municipal) to the highest percentage of 88.0% (Bawku Municipal).

About the frequency of regulatory visits, the number of OTCMS facilities that had received regulatory visits in less than 1 year was very low across the selected MDA’s. A total number of 24 facilities (12.9%) had never received a regulatory visit and the percentage of facilities never inspected was low and varied slightly across the 8 MDA’s.

For interactions in the regulatory environment, the most commonly reported interaction was respondents’ perception that it was important to establish close relationships with inspecting officers. While the percentage of main staff/respondents that had ever received prior notification before regulatory visits was relatively high, the same could not be said either for those who had ever contributed in cash or kind directly or through other(s) to establish relationships or for any other reason.

Location stratification found urban facilities to be more compliant with selected regulation than rural facilities. Most urban OTCMS facilities were seen to have a good state of repair, durable and easily cleanable floor, more staff trained in the past year, a high number of reference books on-premises, medicine supplier’s invoices and receipts retained on the premises, and appropriately mounted signboard compared to rural OTCMS facilities.

Though more staff in urban facilities knew the name of the law that regulated pharmacy practice than staff in rural facilities, the variation was not substantial (1.2% versus 2.0%, respectively). Urban and rural OTCMS facilities were also found to be comparable in terms of availability of owner(s) of facilities on the premises, appropriateness of the size and the design of the dispensing area, stocking of herbal remedies as well as household items.

In terms of frequency of regulatory visits, more urban facilities had received regulatory visits in less than 12 months compared to those in rural locations. Comparing the frequency of regulatory visits in terms of no regulatory visits, there was statistically significantly (*p* = 0.001) more 'no regulatory visits' in rural areas (21.2%) than in urban areas (5.9%).

### Predictors of regulatory compliance of over-the-counter medicine seller facilities

Only 29.2% of facilities were regulatory compliant. Table 4 shows the bivariate and multiple logistic regression analyses of the regulatory compliance. Bivariate logistic regression analysis found staff trained in less than 1 year, OTCMS facility location, prior notification before regulatory visits by inspection officers, and ever made contribution in cash or kind to be predictors of the regulatory compliance (*p* < 0.05, for all; see Table 4). After statistical adjustment, multiple logistic regression analyses found OTCMS facility location (AOR = 4.2, 95% CI 1.74–10.17, *p* = 0.001) and staff trained in less than 1 year (AOR = 2.78, 95% CI 1.02–7.62, *p* = 0.046) were significantly associated with regulatory compliance. Urban OTCMS facilities had four times increased odds of being regulatory compliant. Similarly, facilities that had a main staff trained in less than a year had about three times increased odds of being regulatory compliant.

**Table 4:** Bivariate and multiple logistic regression analyses of factors associated with regulatory compliance

Predictors	Regulatory compliance		Bivariate regression			Multiple regression		
	Non-compliant (*N* = 109)	Compliant (*N* = 45)	OR	95% CI	*p*-value	AOR	95% CI	*p*-value
	*n* (%)	*n* (%)						
Facility location								
Rural	54 (85.7)	9 (14.3)	Ref			Ref		
Urban	55 (60.4)	36 (39.6)	3.93	1.73–8.93	0.001	4.2	1.74–10.17	0.001
Knows of name of law of pharmacy law								
No	105 (72.4)	40 (27.6)						
Yes	3 (100.0)	0 (0.0)			0.999			
Qualified personnel on premises								
No	3 (100.0)	0 (0.0)						
Yes	106 (70.2)	45 (29.8)			0.999			
Staff trained in less than a year								
No	40 (87.0)	6 (13.0)	Ref			Ref		
Yes	69 (63.9)	39 (36.1)	3.77	1.47–9.68	0.006	2.78	1.02–7.62	0.046
Ever received prior notification								
No	54 (79.4)	14 (20.6)	Ref			Ref		
Yes	55 (64.0)	31 (36.0)	2.17	1.04–4.53	0.038	1.28	0.53–3.08	0.587
Ever made contribution in cash or kind								
No	74 (75.5)	24 (24.5)	Ref			Ref		
Yes	35 (62.5)	21 (37.5)	1.85	0.91–3.76	0.090	1.73	0.71–4.23	0.229
Regulatory visit done								
No	4 (100.0)	24 (24.5)						
Yes	105 (70.0)	45 (30.0)			0.999			
Owner of the facility available								
No	71 (74.0)	25 (26.0)	Ref					
Yes	38 (65.5)	20 (34.5)	1.5	0.74–3.03	0.266			

OR, Odds Ratio; AOR, Adjusted Odds Ratio; CI confidence interval; Bivariate logistic regression—Wald test *p*-value of < 0.2 considered for inclusion in the multivariate model; Statistical significance set at *p* ≤ 0.05; Regulatory compliance is a composite variable of three indicators for regulatory practices (retention of medicine supplier’s invoices and receipts on-premises), licensing and registration requirements (appropriate signage), and equipment and material requirements (availability of reference material)

## Discussion

The regulatory compliance of OTCMs facilities using selected regulation as prescribed under the legal and pharmaceutical regulatory framework of Ghana was assessed in this study. Overall, regulatory compliance was low (29.2%). The predictors of regulatory compliance were OTCMS facility location and staff trained in less than 1 year.

Urban facilities were discovered to be more compliant to selected regulation compared to their rural counterparts, although overall compliance was low. The rural facilities were perhaps less compliant due to multiple reasons including lack of infrastructure. Most rural OTCMS facilities are in remote areas with bad road network [31] and this tends to limit the frequency of regulatory visits from personnel of the Pharmacy Council, implying that their activities are not adequately monitored to ensure compliance with the law. Furthermore, the lack of adequate funding/capital could also account for the low compliance of rural OTCMS facilities to laid down regulation for structure and premises, equipment and material, as well as registration and licensing requirements.

In Ghana, OTCMS facilities are required by law to meet stipulated requirements for structure or premises. Compliance with appropriate dispensing area size and design of the dispensing area was found to be high. However, compliance to characteristics relating to the state of repair and the nature of floor surfaces of premises was relatively low, with rural facilities found to have deviated more from this provision than urban facilities. This observation is similar to that obtained from a study in Sri Lanka which found pharmacies in rural dwellings to be more non-compliant to premises and structural requirements [25].

Certified OTCMS practitioners (or the legal owners of the facilities) are required by regulation to be available on the premises any time the facility is open to the public in Ghana. However, our findings showed that only a third of the surveyed facilities had the owner available on the premises, with owner availability below 53%. This is similar to a study in Kenya, East Africa, where the availability of specialized drug shop owners across two selected districts was around 55% [32]. The term “owner” as used in the study in the Upper East Region of Ghana includes those who had been duly registered or certified by the Regulator and those who owned such facilities, but had not been certified by the Pharmacy Council. This implies that the actual percentage of registered practitioners interviewed on various premises during the survey might thus be an underestimation. However, most of these owners encountered in urban facilities in this study were contrary to that observed from studies in Kenya and Nigeria [26, 33] where owners or relatives of owners working as staff were much more likely to be encountered on the premises of rural facilities.

The law requires an OTCMS to possess at least either a Senior Secondary School Certificate (SSSCE), a West African Senior High School Certificate (WASSCE) or its equivalent, pass an OTCMS qualifying examination and undertake a day orientation and pre-licensing training after passing the requisite examination [34]. The proposed 3-month training for OTCMS applicants in place of the 1-day orientation training as contained in the current Registration and Licensing Policy of the Pharmacy Council is still yet to be enforced. The current enforceable requirement for being certified as an OTCMS practitioner in Ghana contrasts with what occurs in other settings like Kenya where stringent requirements are in force and have for a long time been debated in different health systems [35–37]. In Kenya, an individual who wishes to operate a retail drug outlet must undergo 4–5 years pre-training while pharmacy interns and pharmacy technologist would need to fulfil 1-year post-training and 3 years pre-training, respectively.

Personnel/staff qualification, however, did not influence compliance unlike the study in Kenya where staff qualification was a predictor of regulatory compliance [32]. This is because the basic requirement for OTCMS practitioners in Ghana is SSCE or WASSCE which is not relevant to the field of practice [34]. Furthermore, only a few of the main staff (1.6%) enrolled in the study knew the name of the law that regulated pharmacy practice in Ghana. This is comparable to a study in Tanzania where only 3% of owners and 8% of dispensers knew the name of the law that governed pharmacy practice [38]. The findings from this study are however dissimilar to a study in Kenya where about 30% of the main staff knew the name of the law that governed the Kenyan pharmaceutical sector [32]. Our study findings could be attributed to the recent legislative and regulatory changes made in the pharmaceutical sector, where the coming into existence of the Health Professions Regulatory Bodies Act, 2013 (Act 857), saw to the repealing of the Pharmacy Act, 1994, which was previously in force [24]. The relatively low number of staff who knew the name of the law that regulates pharmacy practice in Ghana and Tanzania raises some legitimate questions on the purpose of regulatory visits and what they do entail. Support staff and practitioners would ordinarily not comply with a regulatory provision they are ignorant of and it is expedient that Regulatory Officers take ample time to explain the legal basis of regulatory visits during monitoring activities.

All OTCMS facilities in Ghana must by regulation have reference material on the premises [34]. Such reference materials range from training programme manuals, bulletins, Treatment Guidelines, Medicines Information Handbook, National Formularies and other reference books. However, the findings of this study revealed that compliance with this requirement was relatively low and varied not quite substantially across MDA’s and rural–urban locations. Support staff often indicated that such reference materials were with the facility owners and were not kept on the premises. This implies the owners of such facilities do not appreciate the use of reference material and why such materials are required to be kept on the premises. As anticipated, more urban facilities had reference books compared to rural facilities, and this could be explained by the fact that such reference materials are mostly advertised either in the offices of the regulator (located in Regional capitals) and or during training programmes which most often are held in urban centres and predominantly attended by urban practitioners. The cost of such reference materials as well as the total cost involved in participating in training programmes by rural facility staff could also have been a contributory factor for the low usage or availability of reference materials in the rural facilities.

Another important finding of the study was that OTCMS practitioners did not only stock orthodox drug products, but also herbal remedies and non-drug items such as cotton wool, bandages, sanitary pads among others. The increasing demand for unorthodox or herbal medicine in Ghana could have accounted for the increased availability of herbal drugs across MDA’s and rural–urban locations. Other attributable reasons include increased commercial advertisement of herbal products on airwaves, relatively cheaper cost compared to orthodox medicines as well as the increased public perception that herbal remedies are relatively safer. Household items were interestingly not encountered on any premises. This finding is in agreement with studies in other African countries where about 66% of patent medicine sellers in Nigeria and about 75% of pharmacies in Somalia stocked only drug-related products [3]. The observation is however contrary to another study in Nigeria that reported patent medicine vendors stocked not only food items but also cosmetic products [39]. OTCMS facilities surveyed were found to stock prescription-only medicines and pharmacy medicines in addition to the legally prescribed over-the-counter drugs or Class C drugs. The stocking of prescription-only medicines was very high and comparable across MDAs and rural–urban locations. This is comparable to a study in Nigeria where patent medicine sellers were reported to sell prescription-only drugs in the form of prescription-only analgesics and prescription-only antimalarials, and only 13% of respondents were of the view that that specific regulation was being strictly adhered to [39].

In Ghana, all medicine outlets are required by regulation to retain copies of drug sourcing documents on premises. The retention of medicine supplier’s invoices and receipts as revealed by the study was relatively low and varied across MDA’s and across rural–urban locations. This phenomenon concords with an observation made in Kenya where only 42% of specialized drug shops (SDS) retained prescription records on the premises [32]. These two observations could be due to the fact that keeping such documents on the premises would readily give Regulatory officers an idea of the range of products actually available in stock in a particular facility. OTCMS staff mainly conceal unapproved drug classes, unregistered products, injections and or infusions upon receiving a tip-off on impending regulatory visits. Thus, making such invoices available would make their non-compliance to approved drug classes and or approved products easily detectable.

In addition, OTCMS practitioners are required by regulation to quote their registration numbers as seen on their operating licenses on their signboards. Though there is a possibility for unregistered outlets to also mount signboards, this situation is however rare. Compliance with this regulation as revealed by the study was low in some MDA’s and varied from one MDA to another as well as between rural (43.5%) and urban locations (67.3%). This could be explained by the fact the new format for writing signboards was only introduced in the latter part of the year 2016 and as at the time of the survey most of the facilities encountered were still within the grace period given by the Regulator for all OTCMS practitioners to re-write their signboards in conformity with the newly set standards. The low compliance to this indicator is comparable to studies that utilized the display of licenses on-premises such as in Tanzania, where only one-third of facilities had licenses displayed on premises; in Sri Lanka, where 57% of facilities failed to display licenses on premises [2, 25] and in Kenya where only 56% of facilities surveyed had permit licenses displayed on-premises [32].

Furthermore, the number of OTCMS facilities that had received regulatory visits in less than a year was significantly low across the selected MDA’s and a higher proportion of facilities that had received such regulatory visits were, however, located in urban settings. Similarly, a study in Nigeria reported that only a third of patent medicine stores had received regulatory visits over the past 2 years [39]. Different observations were however made in Sub-Saharan Africa. Two separate studies conducted in Kenya, and another in Tanzania reported that a great number of facilities had been inspected over the past 1 year [2, 32]. The low inspection frequency could be explained by financial constraints, human resource and logistic challenges [23]. This study also showed that the frequency of regulatory visits influenced regulatory outcomes. Though studies that have tried to unravel the relationship between the frequency of inspection visits and regulatory compliance are limited in number, most often, low inspection frequencies have been associated with poor regulatory compliance [27].

Majority of the main staff surveyed were of the opinion that it was very important to establish close relationships or rapport not only with inspecting officers, but also with the entire staff of the Regulatory Body (Pharmacy Council). This kind of rapport would not only be built through frequent visitation by main staff of OTCMS facilities to either inspecting officers or the Office of the Regulator, but also through frequent interaction or communication via various means. A significant number of participants reported that they had ever contributed in cash/kind either directly or through others (mostly OTCMS executives) to support inspecting officers and occasionally, to other staff of the Pharmacy Council. With such gestures, OTCMS staff are probably pre-informed of impending regulatory visits by staff of the Regulator, though information on such visits could also be released by the staff of other OTCMS facilities in other places or other individuals due to the easy identification of the official vehicle(s) of the Pharmacy Council. This explains why a significant number of staff confirmed receipt of prior notification before regulatory visits. In order to remedy this situation, the pharmacy council has since time immemorial enacted a law that necessitates OTCMS to attend continuous professional development activities annually in order to be in good standing to practice. There is also a new policy to be rolled out by the Pharmacy Council which would also serve to train and certify OTCMS staff as well as making it mandatory for such staff to continuously gain Professional development. This approach if implemented would ensure that both practitioners and supporting staff are abreast with current trends in pharmacy practice and changes in the law. Such interactions mentioned above often lead to concealment of regulatory violations and also tacit permission. It thus appears that regulatory officials might be aware of the various regulatory violations committed by over-the-counter medicine retailers, yet, still, they tend to overlook them. This occurrence resulted in main regulatory officers indulging in activities that deviated from the statutory mandate of the particular regulatory body. The likely causes of regulatory non-compliance have been reported by a study in Tanzania to include inadequate knowledge, inadequate inspections and or poor regulatory supervision, and tacit permission from regulatory enforcers [25].

OTCMS facilities by regulation have some equipment requirements to meet as a pre-requisite for registration and licensure in Ghana such as counting trays, which have now become obsolete due to less customer demand for loose tablets. For these facilities, weighing scales and other equipment such as refrigerators are only considered as ancillary devices and as such are not mandatorily required to be provided. This contrasts with what pertains to other settings such as Malawi and Kenya where relevant equipment such as a working refrigerator must be available as a pre-requisite for registration and licensure. Even in those settings, studies have recorded low compliance for the availability of such equipment [32, 40]. Compliance with the availability of a refrigerator was however documented to be high in Sri Lanka [25] and Pakistan [41] according to separate studies. The different regulatory positions concerning this equipment requirement could be due to differences in the availability of electricity across nations and continents or the technological gaps across nations and continents [42].

Future research work in this area could concentrate on studies designed to better understand the causes and consequences of regulatory actions of Inspection Officers and OTCMS retailers. Other studies could also consider data collection on factors of regulation that involve client interaction, particularly those that focus on the safety of clients. This would comprise direct observation or the use of mystery clients previously enrolled in assessing the quality of care as well as certain facets of regulatory compliance to identify key regulatory areas that can be easily assessed. To know the degree to which future educational interventions would be potentially beneficial, studies could also be designed to focus on examining the knowledge of regulations in detail in order to estimate the degree to which regulatory non-compliance manifests discordances between knowledge and practice.

## Limitations of the study

It is important to note certain methodological limitations. Although complementary approaches were used to identify facilities to be involved in the survey, it was still possible to miss some facilities on the ground. However, for the number of facilities identified through the census survey (208) to be significantly higher than the number (176) available on the records of the main Regulator, it implies that some facilities might have been captured through the census survey which was not on the records of the Pharmacy Council of Ghana.

The Pharmacy Council of Ghana did not have the correct number of OTMCS facilities due to the following reasons: (1) The database of the Pharmacy Council (Head Office) only includes facilities that have been issued permanent registration (Business operating licenses with registered numbers). For OTCMS, shortlisted applicants go through examinations and interview after which successful applicants are granted temporal approval letters (valid for a 6-month period from date of issue). On this document, they are required to fulfill laid down requirements including payments and final inspection conduction. Successful applicants who fulfill these requirements are allowed to operate while waiting for their permanent registration documents. (2) The Upper East Regional Office was newly created as at the time of our study. This office was carved out of the then Northern Regional Zonal Office. Apart from challenges in accessing all temporary approval documents from the Northern Regional Office, there were practitioners who had fulfilled the necessary requirements but had for several years still not received permanent registration documents due to bureaucracies. This group excluded unregistered facilities (illegal premises) (never registered and those with expired licenses that had been permanently deleted from the Pharmacy Council's database but probably not communicated to the affected practitioners) as well as those who could not fulfill the necessary requirements but were found to be operating such facilities. (3) This phenomenon is not one peculiar to only our study. There is a similar study where the researchers identified a higher number of retail medicine outlets than recorded by the regulator [32]. The Pharmacy council has officers who routinely inspect the OTCMS facilities and their practices. A possible reason for the differences in numbers could be due the fact that some facilities may be operating illegally. These findings could also draw the attention of the regulator to penalize facilities that operate illegally.

Furthermore, a potential source of bias that cannot be overlooked in the study is the relatively higher rate (10.58%) of refusals. The 22 OTCMS facilities that declined to participate in the study for the sole reason of absence of main responsible staff could also be suspected to be non-compliant to prescribed regulation. Another incidence of bias is reporting bias, especially in assessing items on the tool that required verification, for example, staff qualification and staff training. Information bias cannot also be overlooked on the part of the provider as well as misclassification which could have resulted from recall bias. The need to eliminate subjective judgments as much as possible and focus much more on objective indicators, not making observations on on-going consultations and or raise any suspicion the exercise was an inspection rather than a survey might have limited the ability of the study to present a detailed picture of the regulatory environment. It is acknowledged that some prescribed regulations of much higher public health significance than those selected were not included in the study. This was due to resource constraints as well as the need to eliminate subjective indicators as much as possible. Generalization of the findings of this study especially to larger areas should be done cautiously. The Upper East Region is known to have a lower number of OTCMS facilities and is currently ranked ninth out of ten regions in terms of the total number of OTCMS facilities. Larger and densely populated areas are known to have good accessibility to health facilities and demand for health services is much higher which might influence practice [43]. The displaying of licenses was not assessed because original copies of operating licenses were not available during the period of data collection. They are usually submitted to the Pharmacy Council each year between January and March for renewal. There are most often renewal challenges on the part of the regulating body leading to the delayed release of renewed licenses. Renewed operating licenses are typically released to practitioners in the latter part of the third quarter of the year. Hence, it was not possible to rely on photocopies of operating licenses since authentic features (against counterfeit) on operating licenses could not be assessed on such documents. Also, the non-existence of the Health Facilities Regulatory Agency (HEFRA) in the Upper East region, partly accounted for the refusal to incorporate premises license in the study assessment protocol, as these facilities have not yet been issued premises licenses. More so, an inspection of a license or its display does not fully reflect the status of over-the-counter medicine retail outlets since the certified practitioner may not be actively involved in the day-to-day management of the facility. Again, the validity of permits or operating licenses was not assessed because of the possibility of respondents regarding the interview as an inspection activity. This would likely have led to violent and repulsive behaviour from respondents which would have marred the conducive atmosphere required for the study.

## Conclusion

Overall, regulatory compliance was low, particularly across rural locations, where most of the facilities failed to meet laid down provisions regarding practice, staff, and premises requirements. Though the frequency of inspection visits was significantly low, a regulatory visit in less than a year was a predictor of two regulatory outcomes (retention of medicine suppliers’ invoices and receipts and availability of reference materials). Regulatory interactions recorded was relatively low but had a strong influence on regulatory compliance. The factors for regulatory non-compliance were identified to be insufficient knowledge, infrequent inspection visits and tacit permission resulting from complex interactions within the regulatory environment.

The relatively lower compliance of OTCMS facilities in rural areas of the Upper East region of Ghana which is majorly attributed to lack of infrastructure underscores the need for alternative approaches for their regulation. Policymakers are been called on to put in place pragmatic measures in relation to OTCMS facility’s location and regulatory requirements in order to address the inequities in compliance. This would enable the facilities in rural areas to meet regulatory requirements and also improve compliance. OTCMS who infringe the law should be sanctioned in order to deter others from contravening the law.

## Data Availability

The dataset(s) supporting the conclusions of this article is(are) available on request from the corresponding author.

## Abbreviations

CHPS: Community-Based Health Planning and Services
FDA: Food and Drugs Authority-Ghana
HEFRA: Health Facilities Regulatory Agency
MDA: Municipal and District Assembly
MDA’s: Municipal and District Assemblies
OTCMS: Over-the-Counter Medicine Sellers
PC: Pharmacy Council, Ghana
POM: Prescription-only Medicines
SDS: Specialized Drug Shop
WHO: World Health Organization
